# Sex differences in health-related quality of life and psychological distress among colorectal cancer patients: a 2-year longitudinal study

**DOI:** 10.1007/s11764-024-01616-0

**Published:** 2024-05-24

**Authors:** Cynthia S. Bonhof, Belle H. de Rooij, Dounya Schoormans, Dareczka K. Wasowicz, Gerard Vreugdenhil, Floortje Mols

**Affiliations:** 1 CoRPS—Center of Research on Psychological Disorders and Somatic diseases, Department of Medical and Clinical Psychology, Tilburg University, Tilburg, The Netherlands; 2 Department of Research, Netherlands Comprehensive Cancer Organisation (IKNL), Utrecht, The Netherlands; 3 Department of Surgery, Elisabeth-Twee Steden hospital, Tilburg, The Netherlands; 4 Department of Internal Medicine, Máxima Medical Centre, Eindhoven and Veldhoven, The Netherlands

**Keywords:** Colorectal cancer, Health-related quality of life, Psychological distress, General population, PROFILES

## Abstract

**Purpose:**

While sex differences in the incidence and mortality of colorectal cancer (CRC) are well documented, less is known about sex differences in patients’ health-related quality of life (HRQoL) and psychological distress. To enhance patient-tailored care, we aimed to longitudinally examine sex differences in HRQoL and psychological distress among CRC patients from diagnosis up until 2-year follow-up.

**Methods:**

Newly diagnosed CRC patients from four Dutch hospitals were eligible for participation. Patients (*N* = 334) completed questions on HRQoL (EORTC QLQ-C30) and psychological distress (HADS) before initial treatment (baseline), 4 weeks after surgery, and at 1 and 2 years after diagnosis. Also, HRQoL and psychological distress were assessed in a sex- and age-matched reference population.

**Results:**

When directly comparing female (*N* = 126, 38%) and male (*N* = 208, 62%) CRC patients, female patients reported significantly worse HRQoL, such as more insomnia at baseline, worse physical and role functioning 4 weeks after surgery, more diarrhea at 1 year, and more pain and constipation at 2-year follow-up. However, a comparison with the reference population revealed larger differences between patients and reference in males than in females. For example, at 1- and 2-year follow-up, male patients reported significantly worse cognitive and social functioning, more insomnia, and more anxiety compared with a reference population.

**Conclusions:**

Especially male CRC patients reported worse HRQoL and more psychological distress when compared with a reference population.

**Implications for cancer survivors:**

Knowledge of sex-specific differences in HRQoL and psychological distress among CRC patients may help healthcare providers anticipate and appropriately address patients’ unique healthcare needs.

## Introduction

Colorectal cancer (CRC) is a major health concern worldwide. It is currently the second most commonly diagnosed cancer in women, with 866,000 new cases each year, and the third in men, with 1,066,000 new cases each year [1, 2]. Overall, due to earlier diagnosis and improvements in treatment, mortality rates have decreased in the past three decades, but remain substantially higher for males [3]. Possible explanations for the differences in incidence and mortality include both physiological and behavioral factors. For example, sex hormones may play a role, as estrogenic hormone use among women has been associated with a reduced risk for CRC and improved survival [4, 5]. Overall, men also more often engage in lifestyle-related risk factors (e.g., smoking, alcohol consumption) [6], participate less in cancer screening programs [7, 8], and are less likely to seek medical care [9].

Studies have shown that the health-related quality of life (HRQoL) of CRC patients is reduced in the first year after treatment, but—in the absence of disease recurrence or progression—gradually improves during the years thereafter [10–13]. However, while CRC survivors can reach HRQoL levels that are comparable to those of the general population, some of them may continue to face ongoing problems related to the cancer and its treatment, resulting in low HRQoL and high psychological distress (i.e., mood disorders and symptoms of anxiety and depression) [14–16]. In previous studies among CRC patients, differences in HRQoL were found between male and female patients [17–19]. In a longitudinal study among rectal cancer patients from before surgery up until 2-year follow-up, women reported worse HRQoL, including worse global HRQoL, physical functioning, and sleep quality, and more diarrhea and fatigue compared with men [17]. In another longitudinal study, also among rectal cancer patients, male patients reported worse social functioning, but only at 12 months after sphincter-saving surgery [18] compared with women. To date, no longitudinal studies on sex differences in HRQoL have included colon cancer patients. Regarding sex differences in psychological distress, studies among CRC patients have yielded conflicting findings [15, 20], with male CRC patients reporting more distress compared to females in a 5-year longitudinal study [20] and less psychological distress in a longitudinal study among long-term CRC survivors [15].

Sex differences in HRQoL and psychological distress are already present in the general population, with men reporting better functioning and less symptoms and psychological distress compared with women [21, 22]. To account for these inherent differences, several cross-sectional studies in CRC patients have included a comparison with the general population when examining sex differences in HRQoL and psychological distress [13, 23, 24]. For instance, a German study assessing HRQOL 1 year after CRC diagnosis revealed no sex-specific differences in HRQOL between CRC patients and population controls [13], whereas a cross-sectional secondary analyses of a Dutch study showed that in long-term CRC survivors, sex differences were found between CRC patients and a normative population across multiple domains [23]. Specifically, when male and female patients were directly compared, females reported worse physical and emotional functioning and more nausea and vomiting, insomnia, and anxiety. However, when compared with a reference population, symptoms such as fatigue, dyspnea, anxiety, and depression were found to be more frequent in male patients [23].

Given the increasing emphasis on the importance of providing patient-tailored follow-up care in cancer treatment, identifying sex differences in HRQoL and psychological distress is important. To our knowledge, no longitudinal studies on sex differences in HRQoL have been done among colon cancer patients, and there is a lack of longitudinal studies on sex differences in psychological distress among CRC patients. In addition, no longitudinal study has included a reference population. Therefore, the aim of this prospective study among CRC patients is to first examine the course of HRQoL and psychological distress among male and female patients from diagnosis to 2 years thereafter. Secondly, we aim to directly examine sex differences in longitudinal measurements of HRQoL and psychological distress and compare male and female CRC patients to a reference population, to gain better insight into the relative impact on potential impairments in HRQoL and psychological distress.

## Methods

### Setting, participants, and data collection

This study is based on data from the PROCORE study, a prospective population-based study among CRC patients aimed to examine the impact of CRC and its treatment on patient-reported outcomes. Patient inclusion took place between January 2016 and January 2019 in 4 Dutch hospitals. All eligible patients newly diagnosed with CRC as a primary tumor were invited to participate. Exclusion criteria were (1) previous diagnosis with a different carcinoma, except for basal cell carcinoma of the skin, (2) cognitive impairments, and (3) inability to read or write Dutch. The PROCORE study was approved by the certified Medical Ethic Committee of Medical research Ethics Committees United (registration number NL51119.060.14).

Details of the data collection have previously been described [25]. Data was collected through PROFILES (www.profilesregistry.nl), a registry for the physical and psychosocial impact of cancer and its treatment [26]. PROFILES is directly linked to the Netherlands Cancer Registry (NCR) that collects data from all newly diagnosed cancer patients in the Netherlands [27]. Shortly after diagnosis, and before start of initial treatment, eligible patients were invited to participate via their research nurse or case manager. They received an information package containing an information letter, informed consent form, and the baseline questionnaire. Follow-up questionnaires were sent at 4 weeks after surgery (when applicable), 1 year after diagnosis, and 2 years after diagnosis.

### Measures

#### Sociodemographic and clinical characteristics

Patients’ sociodemographic (i.e., age and sex) and clinical (i.e., cancer type, clinical stage, treatment) information was available from the NCR[27]. Comorbidity was assessed with the adapted Self-Administered Comorbidity Questionnaire (SCQ) [28].While the SCQ also includes the comorbidity “depression,” we excluded this comorbidity as this is also one of our outcome measures. Questions on partner status and educational level were added to the questionnaire.

#### Health-related quality of life

HRQoL was assessed with the EORTC QLQ-C30 (Version 3.0) [29]. It contains five functioning scales (i.e., physical, role, cognitive, emotional, and social functioning), a global QoL scale, three symptom scales (i.e., fatigue, pain, and nausea and vomiting), and six single items (i.e., dyspnea, insomnia, appetite loss, constipation, diarrhea, and financial impact). Each item is scored on a four-point Likert scale ranging from 1 (not at all) to 4 (very much), except for the global QoL items, which range from 1 (very poor) to 7 (excellent). Scores were linearly transformed to a 0–100 scale [30]. Higher scores on the functioning scales and global QoL indicate better functioning and QoL, whereas higher scores on the symptom scales indicate more complaints.

#### Psychological distress

Anxiety and depressive symptoms were assessed with the Hospital Anxiety and Depression Scale (HADS) [31]. It consists of 14 items, seven items assess anxiety symptoms (HADS-A), and the other seven assess depressive symptoms (HADS-D). Items are answered on a four-point Likert scale. Total scores for both the anxiety and depressive symptom scale range from 0 to 21 with higher scores representing more anxiety and depressive symptoms.

#### Reference population

Sociodemographics (i.e., age, sex, marital status), clinical data (i.e., comorbidity), and data on HRQoL (EORTC QLQ-C30) and psychological distress (HADS) from the reference population were obtained from CentERpanel, an online household panel representative of the Dutch-speaking population in the Netherlands [32]. Details of the annual data collection have previously been described [33]. For this study, data from 2011 was used. To ensure the independence of observations, one cancer-free member per household (*N* = 1401) was selected. In addition, as participants in the household panel are relatively young compared with the CRC patients, the oldest person of the household was selected. Of this sample, a random sex- and age-matched reference sample was selected (< 30, 30–34, 35–39, 40–44, 45–49, 50–54, 55–59, 60–64, 65–69, 60–64, 65–69, 70–74, 75–80, ≥ 80), reflecting the distribution of the patient sample. This resulted in a final reference sample of 334 panel members (ratio norm population/cancer patients is 1:1).

### Statistical analyses

NCR data on sociodemographic and clinical characteristics enabled us to compare eligible patients and respondents, using *t* tests for continuous variables and chi-squared (or Fisher’s exact) tests for categorical variables. We also compared differences in baseline sociodemographic and clinical characteristics between (1) male and female CRC patients, (2) male CRC patients and the male reference population, and (3) female CRC patients and the female reference population in the same way.

For our main analyses, we first examined the courses of each HRQoL and psychological distress subscale separately among male and female CRC patients using linear mixed models (LMMs), with maximum likelihood estimation and an unstructured covariance matrix with a 2-level structure (i.e., repeated time points [lower level], patients [higher level]). Time was analyzed as a regular categorical predictor with four levels (i.e., four time points). These analyses were adjusted for age, partner status, educational level, tumor type, cancer stage, and number of comorbidities. As cancer stage and cancer treatment are closely related and we want to avoid multicollinearity, we opted to include cancer stage as a covariate, in line with our previous study [34]. Second, differences in HRQoL and psychological distress between male and female CRC patients at each time point were examined similarly, but without time as a predictor. Additionally, differences in HRQoL and psychological distress between (1) male CRC patients and the male reference population and (2) female CRC patients and the female reference population were also examined in a similar way. However, analyses were adjusted for covariates that were available in both patient and norm samples: age, partner status, educational level, and number of comorbidities.

Analyses were performed using SPSS (IBM SPSS Statistics for Windows, Version 24.0 Armonk, NY: IBM Corps USA). A *p* value < 0.05 was considered statistically significant.

## Results

### Patient characteristics

Of the 713 CRC patients invited for the study, 68% (*n* = 483) completed the questionnaire at baseline, 52% (*n* = 374) at 1-year follow-up, and 49% (*n* = 347) at 2-year follow-uA full flow chart of the study has previously been published [25]. Compared with all eligible patients, respondents were significantly younger, more often male, more likely to receive chemotherapy, and less likely to undergo surgery. In addition, they were significantly less often diagnosed with rectosigmoid cancer, they more often had stage III cancer, and less often stage IV cancer (data not shown).

Of the 397 patients who completed the baseline and at least the questionnaire at 1- or 2-year follow-up, 58 patients were excluded as they were previously diagnosed with cancer and/or had already started treatment at time of baseline. Additionally, five patients were excluded because of missing data on our outcome measures. The final sample consisted of 334 patients, 208 males (62%) and 126 females (38%) (Table 1). Male patients more often had a partner compared with female patients. No other differences in sociodemographic and clinical characteristics were found between male and female patients. Several differences were found between CRC patients and the reference population. Compared with the male reference population (*n* = 208), male CRC patients more often had a partner (92% vs. 85%; *p* = 0.02), more often received medium education (62% vs. 52%; *p* = 0.03), and less often received high education (30% vs. 44%; *p* = 0.004). Female CRC patients more often had a partner (73% vs. 50%; *p* < 0.001) and more often received a low education (14% vs. 6%; *p* = 0.03) compared with the female reference population (*n* = 126).

**Table 1:** Baseline sociodemographic and clinical characteristics of colorectal cancer patients, stratified by sex

	Total (*N* = 334)	Men (*N* = 208, 62%)	Women (*N* = 126, 38%)	*p* value
Age (mean, SD)	66.2 (8.9)	66.7 (8.5)	65.5 (9.5)	0.21
Partner (yes)	283 (85%)	191 (92%)	92 (73%)	**< 0.001**
Education level^a^				0.09
Low	33 (10%)	16 (8%)	17 (14%)	
Medium	210 (63%)	129 (62%)	81 (65%)	
High	89 (27%)	62 (30%)	27 (22%)	
Tumor location				0.16
Colon	235 (71%)	142 (68%)	95 (75%)	
Rectum/rectumsigmoid	97 (29%)	66 (32%)	31 (25%)	
TNM stage				0.21
I	102 (31%)	55 (26%)	47 (37%)	
II	99 (30%)	64 (31%)	35 (28%)	
III	125 (37%)	84 (40%)	41 (33%)	
IV	8 (2%)	5 (2%)	3 (2%)	
Tumor differentiation grade				0.51
Well differentiated	2 (1%)	2 (1%)	0 (0%)	
Moderately differentiated	271 (81)	165 (79%)	106 (84%)	
Poorly differentiated	20 (6%)	12 (6%)	8 (6%)	
Unknown	41 (12%)	29 (14%)	12 (10%)	
Surgery (yes)	328 (98%)	204 (98%)	124 (98%)	0.99
Chemotherapy (yes)	104 (31%)	70 (34%)	34 (27%)	0.20
Radiotherapy (yes)	52 (16%)	36 (17%)	16 (13%)	0.26
Number of comorbidities				0.06
0	93 (28%)	60 (29%)	33 (26%)	
1	117 (35%)	80 (39%)	37 (29%)	
≥ 2	121 (37%)	65 (32%)	56 (44%)	

Variables may deviate from 100% due to rounding off. Bold *p* values indicate statistically significance

*SD* standard deviation

^a^Education: low (no or primary school); medium (lower general secondary education or vocational training); high (pre-university education, high vocational training, university)

### Course of HRQoL and psychological distress

**Fig. 1:**
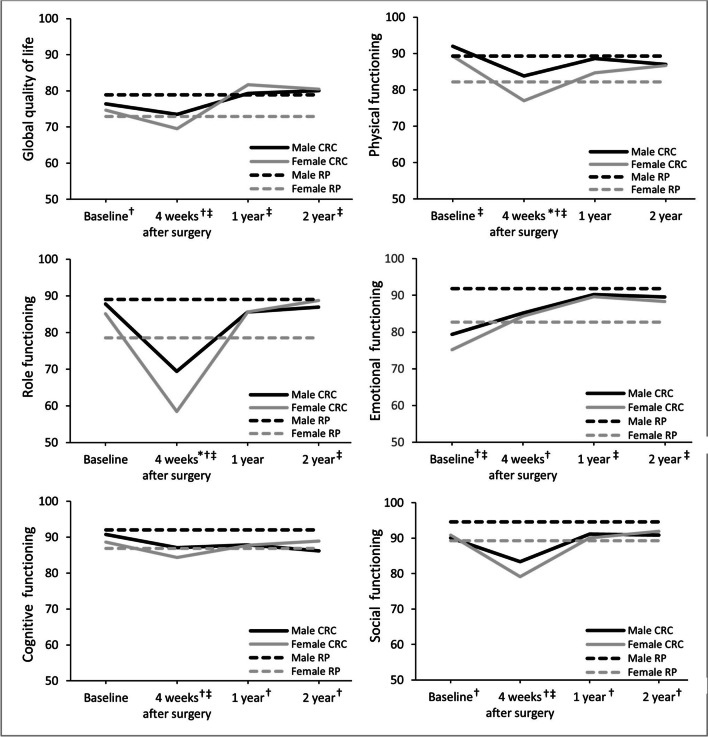
Course of European Organisation for Research and Treatment of Cancer (EORTC) Quality of Life (QoL) Questionnaire Core 30 functioning scores and global QoL for CRC patients and the reference population, stratified by sex. CRC, colorectal cancer; RP, reference population. A higher score on the scales indicates better functioning. The scales in this figure range from 50 to 100 for clear visibility, while scores of the EORTC QLQ-C30 range from 0–100. *Significant difference between male and female CRC patients; †significant difference between male CRC patients and the male reference population, ‡significant difference between female CRC patients and the female reference population

In both sexes, global QoL and all functional scales except for emotional functioning had significantly declined 4 weeks after surgery (Fig. 1). Role and social functioning returned to baseline level at 1-year follow-up, and global QoL increased to a level significantly above baseline. In contrast, physical functioning remained significantly below baseline during the entire follow-up period, while emotional functioning was at its lowest level during baseline and significantly improved during follow-uCognitive functioning remained stable from baseline to follow-up among female patients, but decreased significantly below baseline during the entire follow-up period in male patients.

**Fig. 2:**
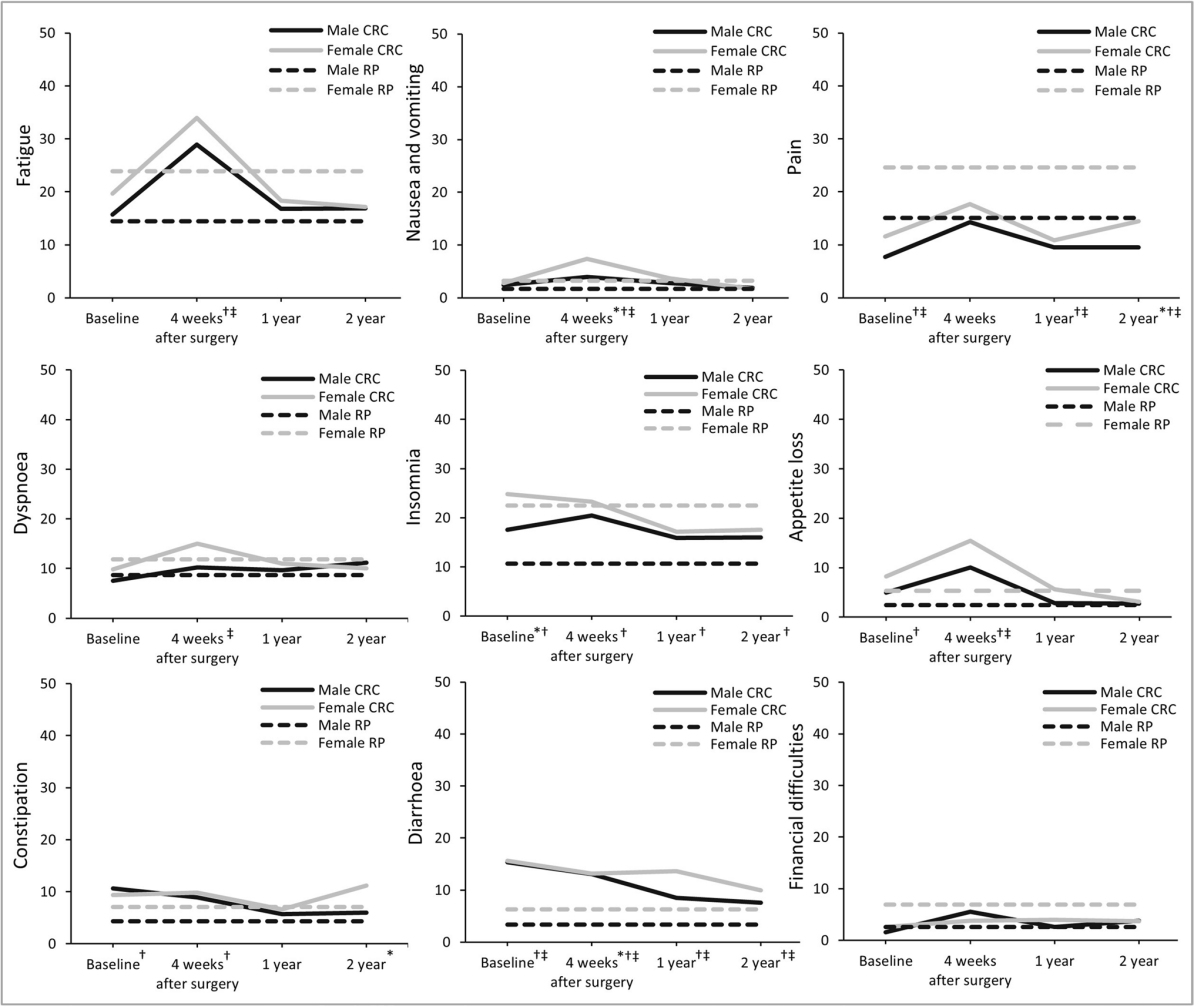
Course of European Organisation for Research and Treatment of Cancer (EORTC) Quality of Life (QoL) Questionnaire Core 30 symptom scores for CRC patients and the reference population, stratified by sex. CRC, colorectal cancer; RP, reference population. A higher score on the scales indicates more symptoms. The scales in this figure range from 0 to 50 for clear visibility, while scores of the EORTC QLQ-C30 range from 0 to 100. *Significant difference between male and female CRC patients; †significant difference between male CRC patients and the male reference population, ‡significant difference between female CRC patients and the female reference population

Regarding symptoms, an increase at 4 weeks after surgery with a return to baseline level at 1-year follow-up was found for fatigue, pain, and nausea and vomiting, with the latter only present in female patients (Fig. 2). Additionally, dyspnea remained stable from baseline to follow-up, and appetite loss increased at 4 weeks after surgery, but significantly decreased to baseline level at 1-year follow-up and below baseline level at 2-year follow-uIn female patients, insomnia was at its highest level during baseline and decreased to below baseline level at 1- and 2-year follow-up, while both insomnia and nausea and vomiting remained stable among males. In male patients, diarrhea and constipation symptoms decreased to below baseline level at 1- and 2-year follow-up, while financial difficulties increased at 4 weeks after surgery, decreased to baseline level at 1-year follow-up, and significantly increased to above baseline level at 2-year follow-uIn contrast, diarrhea, constipation, and financial difficulties remained at baseline level during follow-up among female patients. Finally, anxiety symptoms significantly improved among both sexes at 4 weeks after surgery and remained below baseline during follow-up, while depressive symptoms remained at baseline level.

### Male vs female CRC patients

Compared with male patients, female patients reported more insomnia at baseline, a worse physical and role functioning and more nausea and vomiting at 4 weeks after surgery, more diarrhea at 1-year follow-up, and more pain and constipation at 2-year follow-up (Figs. 1 and 2; Table 2). No other differences in HRQoL or psychological distress were found between the two sexes.

**Table 2:** Course of health-related quality of life and psychological distress among colorectal cancer patients, stratified by sex

	Baseline		Four weeks after surgery		1-year follow-up		2-year follow-up	
	Male (*n* = 208)	Female (*n* = 126)	Male (*n* = 189)	Female (*n* = 114)	Male (*n* = 194)	Female (*n* = 119)	Male (*n* = 185)	Female (*n* = 109)
EORTC QLQ-C30 functional scales, *M* (SD)								
Global Quality of life	76.4 (16.9)	74.7 (20.6)	73.5 (17.9)	69.5 (20.7)	79.3 (17.6)	81.7 (17.5)	80.1 (15.9)	80.5 (17.9)
Physical functioning	92.0 (13.3)	89.3 (14.0)	83.8 (15.6)	77.0 (17.3)**	88.6 (15.3)	84.7 (18.3)	87.0 (17.5)	86.7 (13.9)
Role functioning	87.8 (21.5)	85.1 (26.6)	69.4 (29.4)	58.4 (30.7)**	85.6 (23.6)	85.6 (22.9)	86.9 (24.4)	88.7 (21.6)
Emotional functioning	79.4 (20.1)	75.2 (21.0)	85.2 (17.1)	84.3 (20.4)	90.2 (16.6)	89.7 (17.3)	89.6 (16.6)	88.3 (15.9)
Cognitive functioning	90.8 (15.1)	88.6 (17.7)	87.1 (17.1)	84.4 (18.8)	87.9 (18.7)	87.8 (18.3)	86.2 (20.2)	88.9 (16.0)
Social functioning	90.1 (17.7)	90.9 (16.9)	83.4 (21.5)	79.1 (22.5)	91.2 (16.4)	90.1 (18.9)	90.9 (17.8)	92.0 (16.8)
EORTC QLQ-C30 symptom scales, *M* (SD)								
Fatigue	15.7 (20.4)	19.7 (24.1)	28.9 (24.4)	34.0 (24.7)	16.8 (21.4)	18.3 (22.1)	16.9 (20.2)	17.2 (20.0)
Nausea and vomiting	2.4 (8.5)	2.8 (7.8)	4.0 (11.0)	7.4 (15.7)*	2.8 (10.3)	3.7 (12.7)	1.9 (8.1)	1.7 (6.0)
Pain	7.7 (16.5)	11.6 (19.7)	14.3 (21.4)	17.7 (23.5)	9.5 (18.8)	10.9 (21.6)	9.5 (18.6)	14.5 (22.9)*
Dyspnea	7.5 (17.7)	9.8 (21.5)	10.2 (19.8)	15.0 (22.7)	9.7 (19.5)	11.0 (21.4)	11.2 (21.6)	10.0 (20.1)
Insomnia	17.6 (26.0)	24.8 (26.4)*	20.5 (28.8)	23.3 (29.2)	15.9 (28.7)	17.2 (24.9)	16.0 (26.1)	17.6 (23.0)
Appetite loss	5.0 (14.7)	8.2 (19.6)	10.1 (21.4)	15.5 (23.6)	2.8 (11.9)	5.6 (16.5)	2.7 (13.5)	3.1 (10.7)
Constipation	10.6 (20.4)	9.4 (20.6)	8.9 (21.1)	9.8 (18.8)	5.7 (15.5)	6.6 (14.1)	6.0 (16.2)	11.2 (18.3)*
Diarrhea	15.4 (26.0)	15.6 (23.4)	13.1 (21.9)	13.2 (21.2)	8.5 (18.1)	13.6 (23.2)*	7.6 (17.8)	10.0 (20.6)
Financial impact	1.6 (7.1)	2.6 (9.0)	5.5 (15.4)	3.8 (12.4)	2.6 (9.6)	4.0 (14.6)	3.8 (14.1)	3.7 (16.0)
Hospital Anxiety and Depression Scale, *M* (SD)								
Anxiety symptoms	5.0 (4.0)	5.5 (3.8)	3.4 (3.2)	4.1 (3.7)	3.4 (3.3)	4.3 (3.7)	3.3 (3.3)	4.1 (3.9)
Depressive symptoms	3.8 (3.6)	3.6 (3.6)	3.7 (3.7)	4.0 (3.7)	3.5 (3.4)	3.2 (3.2)	3.8 (3.6)	3.6 (3.4)

A higher score indicates a better functioning (EORTC QLQ-C30 functional scales) or more symptoms (EORTC QLQ-C30 symptom scales and Hospital Anxiety and Depression Scale). Analyses are adjusted for age, partner status, educational level, tumor type, cancer stage, surgery, chemotherapy, radiotherapy, and number of comorbidities

*M* mean, *SD* standard deviation, *EORTC* European Organization for Research and Treatment of Cancer

*p < 0.05, **p < 0.01

### Comparison with the reference population

Both sexes reported most differences at 4 weeks after surgery when compared with the reference population (Figs. 1 and 2; Table 3). Specifically, both male and female patients reported worse global quality of life and worse physical, role, cognitive, and social functioning. Male patients also reported worse emotional functioning compared with the male reference population, while no differences were found in their female counterparts. Furthermore, while male patients reported worse cognitive and social functioning at 1- and 2-year follow-up, female patients reported better global quality of life and emotional functioning at 1- and 2-year follow-up and better role functioning at 2-year follow-up.

**Table 3:** Comparison between cancer survivors and the sex- and age-matched reference population

	Baseline		Four weeks after surgery		1-year follow-up		2-year follow-up	
	Male (*n* = 208)	Female (*n* = 126)	Male (*n* = 189)	Female (*n* = 114)	Male (*n* = 194)	Female (*n* = =119)	Male (*n* = 185)	Female (*n* = 109)
EORTC QLQ-C30 functional scales, *M* (SD)								
Global Quality of life	− 2.5*	+ 1.8	− 5.4***	− 3.4*	+ 0.4	+ 8.8**	+ 1.2	+ 7.6*
Physical functioning	+ 2.7	+ 7.1*	− 5.5***	− 5.2***	**−** 0.7	+ 2.5	**−** 2.3	+ 4.5
Role functioning	**−** 1.2	+ 6.5	− 19.6***	− 20.1***	**−** 3.4	+ 7.0	**−** 2.1	+ 10.1*
Emotional functioning	− 12.4***	− 7.5*	− 6.6***	+ 1.6	**−** 1.6	+ 7.0***	**−** 2.2	+ 5.6**
Cognitive functioning	**−** 1.2	+ 1.7	− 4.9***	− 2.5***	− 4.1**	+ 0.9	− 5.8***	+ 2.0
Social functioning	− 4.5**	+ 1.6	− 11.2***	− 10.2***	− 3.4*	+ 0.8	− 3.7*	+ 2.7
EORTC QLQ-C30 symptom scales, *M* (SD)								
Fatigue	+ 1.2	**−** 4.2	+ 14.4***	+ 10.1***	+ 2.3	**−** 5.6	+ 2.4	**−** 6.7
Nausea and vomiting	+ 0.7	**−** 0.4	+ 2.3*	+ 4.2*	+ 1.1	+ 0.5	+ 0.2	**−** 1.5
Pain	− 7.4***	− 13.0***	**−** 0.8	**−** 6.9	− 5.6**	− 13.7***	− 5.6**	− 10.1*
Dyspnea	**−** 1.2	**−** 2.1	+ 1.5	+ 3.1*	+ 1.0	**−** 0.9	+ 2.5	**−** 1.9
Insomnia	+ 6.9***	+ 2.3	+ 9.8***	+ 0.8	+ 5.2*	**−** 5.3	+ 5.3*	**−** 4.9
Appetite loss	+ 2.6*	+ 2.9	+ 7.7***	+ 10.2***	+ 0.4	+ 0.3	+ 0.3	**−** 2.2
Constipation	+ 6.3***	+ 2.3	+ 4.6**	+ 2.7	+ 1.4	**−** 0.5	+ 1.7	+ 4.1
Diarrhea	+ 12.0***	+ 9.3***	+ 9.7***	+ 6.9**	+ 5.1***	+ 7.3**	+ 4.2**	+ 3.7*
Financial difficulties	**−** 1.0	**−** 4.3	+2.9	**−** 3.1	0	**−** 2.9	+ 1.2	**−** 3.2
Hospital Anxiety and Depression Scale, *M* (SD)								
Anxiety symptoms	+ 2.4***	+ 1.0	+ 0.8**	**−** 0.4	+ 0.8*	**−** 0.2	+ 0.7*	**−** 0.4
Depressive symptoms	+ 0.3	**−** 0.5	+ 0.2	**−** 0.1	0	**−** 0.9	+ 0.3	**−** 0.5

A higher score indicates a better functioning (EORTC QLQ-C30 functional scales) or more symptoms (EORTC QLQ-C30 symptom scales and Hospital Anxiety and Depression Scale). A negative value indicates that CRC patients reported a lower score compared with the sex- and age-matched reference population, while a positive value indicates that CRC patients reported a higher score compared with the sex- and age-matched reference population. Analyses are adjusted for age, partner status, educational level, and number of comorbidities

*M* mean, *SD* standard deviation, *EORTC* European Organization for Research and Treatment of Cancer

*p < 0.05, **p < 0.01, ***p < 0.001

Regarding symptoms, both sexes reported less pain but more diarrhea at baseline compared with the reference population, while only male patients reported more insomnia, appetite loss, and constipation (Table 3). At 4 weeks after surgery, both sexes reported more fatigue, nausea and vomiting, appetite loss, and diarrhea. In addition, male patients reported more constipation and insomnia, and female patients more dyspnea compared with the reference population. At 1- and 2-year follow-up, both sexes reported less pain but more diarrhea, while male patients still reported more insomnia. Finally, male patients reported more anxiety symptoms at all time points, while no differences were found among females.

## Discussion

In this prospective study among CRC patients, we first showed that, among both sexes, HRQoL was generally reduced at 4 weeks after surgery but had improved at 1- and 2-year follow-uSome outcome measures improved to a level significantly better than baseline, such as global quality of life and anxiety symptoms. In contrast, physical functioning remained significantly below baseline during follow-uThese results are consistent with other studies among CRC patients which showed that HRQoL is reduced in the first year after treatment, but—in the absence of disease recurrence of progression—gradually improves during follow-up [10–13].

Comparing female CRC patients directly to male CRC patients showed that female patients reported more insomnia at baseline, worse physical and role functioning and more pain 4 weeks after surgery, more diarrhea at 1-year follow-up, and more pain and constipation at 2-year follow-uWhen compared with a sex- and age-matched reference population, more sex differences came to light. Interestingly, it was actually the *male* patients who reported worse functioning and more symptoms when compared with the male reference population. For example, male patients reported worse cognitive and social functioning, and more insomnia, diarrhea, and anxiety during the entire follow-up period. Interestingly, female patients reported a *better* global QOL, emotional functioning, and role functioning during follow-up compared with the female reference population.

The finding that female patients reported worse functioning and more symptoms when directly compared with male patients is in line with a study among rectal cancer patients from before surgery up until 2-year follow-up in which women reported worse HRQoL, including worse global HRQoL, physical functioning, and sleep quality, and more diarrhea and fatigue compared with men [17]. In our study, the comparison with a reference population resulted in different findings than the direct comparison between male and female patients: a worse functioning and more symptoms were reported more frequently by male patients than female patients when compared with the reference population. These findings are in contrast with a cross-sectional study among CRC patients 1 year after diagnosis, in which no sex differences were found when compared with a reference population [13]. However, our findings are in line with a cross-sectional study among long-term CRC patients in which female patients reported worse physical and emotional functioning and more nausea and vomiting, insomnia, and anxiety when directly compared with male patients, while symptoms such as fatigue, dyspnea, anxiety, and depression were more frequent in male patients when compared with a reference population [23].

The results of our study first show that a comparison with a reference population is very valuable when assessing sex differences in HRQoL and psychological distress among CRC patients. As sex differences in HRQoL and psychological distress are already present in the general population, with men reporting better functioning and less symptoms and psychological distress compared with women [21, 22], a direct comparison between male and female CRC patients does not adequately capture sex differences in HRQoL and psychological distress. In addition, as we seek to provide patient-tailored and personalized (follow-up) care, the found sex differences in HRQoL and psychological distress should not be ignored. First, appropriate (sex-specific) information provision provided by healthcare professionals or via websites or flyers may help patients feel more confident in dealing with their health-related issues and may help them better adjust to symptoms [35, 36]. Second, knowledge of sex-specific differences in HRQoL and psychological distress among CRC patients may help health care providers anticipate and appropriately address patients’ unique healthcare needs. Particularly male CRC patients seem to be in need of (sex-specific) follow-up care, as they especially reported worse HRQoL and more psychological distress when compared with the reference population. However, men are generally less likely to seek help for physical and mental difficulties [37]. This might be one of the reasons why the recovery of HRQoL and psychological distress takes longer after diagnosis and treatment among males compared to females. Methods to both provide information and diminish effects on HRQoL and psychological distress should thus be explored in a sex-specific way.

It is not completely clear why the worse functioning and more symptoms found among male patients in comparison with a reference population was, aside from more diarrhea, not found among female patients and why female patients reported better global quality of life and better role and emotional functioning compared with the reference population. Post-traumatic growth, a positive psychological change that can occur from a struggle with a highly challenging adverse life event like cancer [38] may be a possible explanation as studies in cancer and other traumatic events suggest that women have higher levels of post-traumatic growth than men [39, 40]. Reasons behind this difference are not fully understood, but could be due to differences in coping, appraisal of stressor, or cultural expectations [41, 42].

Several limitations should be acknowledged. First, eligible patients and respondents differed in some sociodemographic and clinical characteristics. Generalization of our results to the general population of CRC patients should thus be done with caution. Second, respondents of observational studies generally have a better survival, and their HRQoL may be systematically higher compared with non-participants, and therefore, they represent a healthier population [43]. In addition, it is unclear why patients were lost to follow-uIt could be that patients stopped participating in our study due to a poor HRQoL or a high level of psychological distress [44]. Both limitations could have led to an underestimation of the results in male CRC patients and an overestimation of the results in female CRC patients.

Despite these limitations, this study is, to our knowledge, the first prospective study to show sex differences in HRQoL and psychological distress among patients with CRC in comparison with a reference population. A major strength of this study is the comparison with a sex- and age-matched reference population, which revealed sex differences in HRQoL and psychological distress that otherwise would not have been observed. Other strengths are the prospective design and the baseline measurement prior to treatment which enabled us to examine sex-specific changes in HRQoL and psychological distress over time and to examine whether these changes return to baseline level after treatment, or even improve beyond that.

In conclusion, this prospective study among CRC patients from diagnosis up until 2-year follow-up showed sex-specific differences in HRQoL and psychological distress. Although overall female patients reported worse functioning and more symptoms compared to male patients, male patients reported worse HRQoL and more psychological distress when compared with a reference population. As we seek to provide patient-tailored and personalized (follow-up) care, knowledge of sex-specific differences in HRQoL and psychological distress among CRC patients should be utilized to help health care providers anticipate and appropriately address patients’ sex-specific healthcare needs. Methods to both provide information and diminish effects on HRQoL and psychological distress should be explored in a sex-specific way.

## Data Availability

The data that support the findings of this study are available upon request from the profiles registry (www.profilesregistry.nl).

## Abbreviations

CRC: Colorectal cancer
HADS: Hospital Anxiety and Depression Scale (HADS)
HRQoL: Health-related quality of life
LMM: Linear mixed models
NCR: Netherlands Cancer Registry
PROFILES: Patient Reported Outcomes Following Initial Treatment and Long Term Evaluation of Survivorship
SCQ: Self-Administered Comorbidity Questionnaire
